# An Examination of Occupational Therapy Telehealth Service Delivery Among Novice Users During the COVID-19 Pandemic

**DOI:** 10.5195/ijt.2023.6544

**Published:** 2023-05-11

**Authors:** Lori E. Breeden, Hannah T. Tyger, Alexandra M. Reckers, Megan Johnson, Analicia M. Morales, Lauren Ober, Mackenzie A. Williams

**Affiliations:** University of Indianapolis, School of Occupational Therapy, Indianapolis, Indiana, United States

**Keywords:** Client champion, Coaching, Occupational therapy, Telehealth

## Abstract

The COVID-19 pandemic allowed for widespread implementation of telehealth as a delivery method for occupational therapy (OT) services. The purpose of this study was to investigate the perceptions of novice telehealth OT practitioners regarding telehealth as a delivery method for OT services. Quantitative data was collected through a modified version of the Telehealth Usability Questionnaire and analyzed via descriptive statistics. Qualitative data was collected by open-ended questions and analyzed via thematic analysis. OT practitioners' responses revealed four major themes: logistics of telehealth practice, role of client champions, capacity of the OT practitioner, and styles and approaches. The study revealed that OT sessions delivered via telehealth increased access to clients and continuity of services. Client champion engagement, effective coaching strategies, and practitioner flexibility supported the success of OT telehealth sessions.

With the onset of the COVID-19 pandemic in 2020, use of telehealth as a delivery method for OT services expanded. Telehealth was usable for practice in less than 30 states in the US before the COVID-19 pandemic (Bierman, et al., 2018). Access to telehealth services increased with the COVID-19 pandemic and public health emergency. Immediate action was needed, and government agencies alongside private insurance companies worked to ensure OT services could be provided via telehealth (American Occupational Therapy Association, 2022). Permanent legislation has not yet reached every state, and legislation beyond the end of the public health emergency is necessary for the continuation of telehealth services (American Occupational Therapy Association, 2022). Quickly evolving legislation of this delivery format during a health emergency has resulted in the use of telehealth by many practitioners who were novice users.

Traditionally, occupational therapy (OT) practitioners utilize telehealth to provide access to therapy when traditional services are not feasible (Renda & Lape, 2018). In the states allowing the use of telehealth as a delivery method prior to the pandemic, occupational therapists work with clients in environments that are familiar and provide interventions that translate to their daily lives (Cason, 2015). Shulver et al. (2016) found that clinicians had positive outlooks on the potential of providing OT services to a wider population of clients using telehealth, especially in more rural areas where clients may have limited access to health care resources. This positive practitioner outlook is an important step to establishing and sustaining telehealth as an OT service delivery method.

Wade et al. (2014) developed a theory on the uptake and sustainability of telehealth practice and identified clinician acceptance as the most important element in adoption of this service delivery method. This model indicates that a positive attitude, adequate technology, and good relationships between providers influence the willingness of a clinician to use telehealth. Through the lens of this theory, health care organizations should consider the perceptions of telehealth practitioners, which may be strongly influenced by their experience during the COVID-19 pandemic. Therefore, it will be important to understand factors that foster positive regard for telehealth as it becomes a more widely used delivery method for OT services.

The purpose of this study was to investigate the perceptions of OT practitioners, who were novice telehealth users during the COVID-19 pandemic, and their experiences of telehealth as a delivery method for OT services. The principal questions that guided the study were:

- What are the perceptions of occupational therapy practitioners who were novel telehealth users on the quality of telehealth as an occupational therapy delivery method?
  - How did the telehealth delivery method support or hinder the provision of OT services?
- What are the perceptions of occupational therapy practitioners who were novel telehealth users on the usability of telehealth as an occupational therapy delivery method?

## Methods

### Study Design

The study, a mixed methods design, utilized a modified version of the Telehealth Usability Questionnaire (TUQ) to quantitatively assess OT practitioner perceptions of usability of telehealth (Parmanto, et al., 2016). Open-ended follow up questions to the TUQ were administered to collect qualitative data about aspects of OT practice that were supported or hindered by telehealth as a delivery method.

### Participants

Twenty-three licensed OT practitioners who were novel telehealth users during the time of the COVID-19 pandemic were included in this study. OT students, or practitioners with more than one year of telehealth experience prior to the COVID-19 pandemic were excluded from the study.

### Measures

This study utilized a modified version of the TUQ survey instrument. Per the authors of this tool, “the TUQ utilizes questions that can be modified to correctly address the participants (clinicians or patients) and the telehealth system” (Parmanto, et al., 2016, p. 4). Our modification included changing items on the instrument to measure perceptions of OT practitioners on the usability of telehealth via Likert scale (Parmanto, et al., 2016). Participants rated each question on their level of agreement with the statement using a scale from 1 (*disagree)* to 7 *(agree)*. Subscales of usefulness, ease of use, effectiveness, reliability, and satisfaction were found to be internally consistent and were used to guide the question creation in the original TUQ (Parmanto, et al., 2016). Table 1 contains the modified TUQ questions that were used in our survey.

**Table 1:** Modified Telehealth Usability Questionnaire

Question	Subscale
Usefulness	
1	Telehealth improves my client's access to healthcare services.
2	Telehealth saves my client's time traveling to a hospital or specialist clinic.
3	Telehealth provides for my client's healthcare needs.
Ease of Use	
4	It was simple to use this system.
5	It was easy to learn to use the system.
6	I believe I could become productive quickly using this system.
7	The way I interact with this system is pleasant.
8	I like using the system.
9	The system is simple and easy to understand.
Effectiveness	
10	This system is able to do everything I would want it to be able to do.
11	I could easily talk to the clinician using the telehealth system.
12	I could hear the clinician clearly using the telehealth system.
13	I felt I was able to express myself effectively.
14	Using the telehealth system, I can see the clinician as well as if we met in person.
Reliability	
15	I think the visits provided over the telehealth system are the same as in-person visits.
16	Whenever I made a mistake using the system, I could recover easily and quickly.
17	The system gave error messages that clearly told me how to fix problems.
Satisfaction	
18	I feel comfortable communicating with the clinician using the telehealth system.
19	Telehealth is an acceptable way to receive healthcare services.
20	I would use telehealth services again.
21	Overall, I am satisfied with this telehealth system.

*Note.* System refers to telehealth as a delivery method for OT services.

The survey concluded with open-ended follow-up questions which warranted written responses to assess how OT services are supported or hindered by using telehealth as a delivery method. The following questions were asked to gain more insight into OT practitioner perceptions on telehealth as a delivery method:

- How did the telehealth platform support or hinder the administration of OT evaluations?
- How did the telehealth platform support or hinder the delivery of OT interventions?
- Did you perceive any population specific differences (i.e., age, diagnostic group) in the practicality of using telehealth as a delivery method for occupational therapy services?
- Do you perceive any intervention specific differences (i.e., therapeutic activities and exercise, Activities of Daily Living (ADL) training, neuro-reeducation) using telehealth as a delivery method for occupational therapy services?
- How did telehealth change the way you work?
- Do you see OT service delivery via telehealth as an important part of your practice moving forward? If so, how will you use it?

An expert in the field of occupational therapy delivered via telehealth recommended the use of the modifiable TUQ survey to help ensure construct validity of the study. The open-ended follow-up questions devised were also assessed by the expert to ensure content validity.

### Procedure

Recruitment of survey participants occurred by way of a posting in AOTA's online community, *CommunOT*, state OT association resources, and occupational therapy Facebook groups. The TUQ survey was administered using Qualtrics XM © software (2021) to OT practitioners who were novel telehealth users. The survey was open for eight weeks, sending reminders once a week (Andrews, et al., 2005).

### Data Analysis

The quantitative data used descriptive statistics to analyze the views of novel telehealth users on the usability of telehealth as an OT service delivery method. Descriptive statistics were calculated for the dimensions of usefulness, ease of use, effectiveness, reliability, and satisfaction of telehealth delivery systems.

The qualitative data collected from the open-ended questions was analyzed using Braun and Clarke's approach to simple thematic analysis. Simple thematic analysis is, “a method for identifying, analyzing, organizing, describing, and reporting themes found within a data set” (Braun & Clarke, 2006, p. 79). The six phases to simple thematic analysis include “Phase 1: familiarizing yourself with your data, Phase 2: generating initial codes, Phase 3: searching for themes, Phase 4: reviewing themes, Phase 5: defining and naming themes, and Phase 6: producing the report” (Braun & Clarke, 2006, p. 87).

Trustworthiness of the study was ensured by triangulation of collected data and corroboration of current evidence. Additionally, researchers maintained an audit trail throughout the qualitative analysis to ensure confirmability of the data.

### Data Protection

No identifying participant information was collected from survey responses or connected to survey results. The data collected from the Qualtrics-issued survey was stored on a password-protected computer that remained stored in a locked office.

## Results

### Quantitative Data

Table 2 shows the means and standard deviations of participant perceptions on the usability, ease of use, effectiveness, reliability, and satisfaction of telehealth as a delivery method for OT services. Participants had the most positive perceptions of the usefulness of telehealth, indicated by having the highest mean. Participants had the most negative experiences with the reliability of telehealth, indicated by having the lowest mean. Overall, the means are centralized, and the standard deviations indicate high variance showing that many of the participants had polarized views on telehealth as a delivery method for OT services.

**Table 2:** Descriptive Statistics

Dimensions	Mean	Standard Deviation
Usefulness	5.12	1.326666667
Ease of Use	4.586666667	1.5
Effectiveness	3.968	1.546
Reliability	3.173333333	1.573333333
Satisfaction	4.74	1.49

### Qualitative Data

The responses to the open-ended questions revealed four major categories related to OT practice via telehealth: (a) logistics of telehealth practice, (b) role of client champions, (c) capacity of the OT practitioner, and (d) styles and approaches.

#### Logistics of Telehealth Practice

Logistically, OT practice via telehealth is supported through increased access to services, continuity of care, improved efficiency, and the ability to visualize the home environment. Participant 14 commented “As a part of a service when either practitioner or client is unable to meet in person due to injury, illness, or inconvenient timing. Telehealth is more flexible and allows for other times that may not be able to be seen in person for sessions.” Participant 3 shared that telehealth improves timeliness of service, stating “the patients are able to start their therapy services sooner.” Participant 2 shared that telehealth allows OT practitioners “to see the person in their home environment and to help them with set up.”

Participants also shared various logistics of telehealth practice that created barriers to successful service delivery, including technical difficulties, distribution of materials, and the nature of the virtual environment. Technical difficulties included “internet issues” (Participant 15), “controlling background noise” (Participant 5), and clients not being “computer literate” (Participant 2). Many participants mentioned that “It was hard to ensure the client had the needed materials” (Participant 20), and if clients needed materials, they “needed to come in to get them” (Participant 2) or “needed to be sent prior to OT” (Participant 12).

There are several challenges created by the nature of the virtual environment, especially overcoming the inability to be hands-on, difficulties visualizing the client, or struggling to demonstrate activities properly. Multiple participants stated that one of the largest barriers was “not being able to provide hands-on intervention” (Participant 22) and “not being able to physically cue and correct mechanics of movement” (Participant 2). Participant 2 also stated that “it was often difficult to see what some clients were actually doing,” while Participant 10 shared that it was “harder to demonstrate certain activities and tasks.”

#### Role of Client Champions

Client champions refer to the caregiver, parent, or support person helping the client within their environment during a telehealth session. Client champions, engagement and ability level are two influential components to the success of OT services delivered via telehealth. Participant 19 stated that pediatric settings “required the parent to assist with all aspects of treatment,” and Participant 14 shared that “clients with parents willing to participate in sessions benefitted the most.” According to Participant 7, the effectiveness of the session was “dependent on the quality of [the] support present in [the] person on the other end of the line.”

Communication developed between OT practitioners and client champions via telehealth can create positive changes during in-person services. Participant 14 commented that telehealth practice “changed the way I interacted with clients online and in-person and their families regarding language used to describe strategies for intervention,” and that they experienced “increased parent coaching and parent involvement in the session once met in-person.”

#### Capacity of the OT Practitioner

OT practitioners used their clinical skills to adjust their therapeutic approach for the client when delivering a session via telehealth. OT delivered via telehealth required “more flexibility” (Participant 1) and OT practitioners had to be “more creative” (Participant 10). OT practitioners adapted the language they used to communicate with the clients and client champions during telehealth sessions. Participant 22 claimed that services via telehealth made them “better at communicating strategies to parents” and “more resourceful with interventions.” Participant 3 identified that they used their clinical knowledge to “upgrade or downgrade sessions quickly and reevaluate based on the patient presentation” during telehealth sessions.

OT practitioners discussed the importance of acting as a coach during telehealth sessions. Participant 7 shared that “when you can't be there, you have to hand over that control and really coach,” and that the success of the session “is reliant on the ability of the OT to coach the client and carer.” Participant 14 noted that OT practice via telehealth was supported using “parent education and coaching models.” Through the implementation of coaching skills, clients can experience increased self-efficacy in their abilities. Participant 7 described “the capacity of the clients and their support network to take an increased ownership of activities, tasks, games, practice opportunities and strategies strongly influences the difference in success experienced.” For example, Participant 7 claimed “it can be a really powerful way to empower and upskill parents/carers and boost their confidence as their child's first educator.”

#### Styles and Approaches

Participants in this study identified styles and approaches to delivering OT services via telehealth that supported or hindered the success of the session. Several participants mentioned the assessment style chosen affected successful OT evaluation via telehealth. Participant 10 noted that “verbal interviews and assessments were easy to administer.” Participant 3 shared that they “like to use assessments that have objective numbers and can be easily reassessed.”

Participants shared several specific intervention ideas that were successful during telehealth OT sessions. When asked about interventions utilized, participants shared that “education via telehealth seems to work well” (Participant 10), “therapeutic exercise or therapeutic activity [interventions] were most practical” (Participant 23), and “working on visual perceptual skills, handwriting, feeding skills were great treatment uses for telehealth.” (Participant 12). Participant 18 suggested that telehealth necessitates a more creative approach to intervention sessions, and “made me think of more basic activities like using a washcloth or dish towel to work on hand strength.”

Participants disagreed about the effectiveness of ADL interventions performed via telehealth. Participant 4 shared that “ADL and home related occupations were easier to address via telehealth,” while Participant 9 noted that “ADL training is hard.” Participant 23 shared that “dressing can easily be done over clothes” but “toileting/bathing aren't practical.” Participant 10 spoke about the difficulty of engaging the clients, stating “it's harder to engage fully in therapeutic activities and definitely not the same for group work and social skills training.”

## Discussion

When in-person services were not available, OT practitioners valued the use of the virtual platform to access clients via telehealth. Practitioners indicated that telehealth supported increased service continuity and efficiency, and increased access to populations which allowed them to continue OT services when in-person services were unavailable. This aligns with findings from Tenforde et al. (2020) that telehealth increased continuity of care within the home environment, and efficiency for OT practitioners and clients as virtual platforms eliminated travel time and increased convenience. Given that the nature of the virtual environment prevents the use of hands-on interventions, OT practitioners must develop alternative approaches to connect with their clients and facilitate skill development.

Several participants expressed that OT delivered via telehealth was more effective when client champions participated and were engaged during sessions. Similarly, Gately et al. (2022) found that OT practitioners using telehealth felt that caregiver involvement during sessions improved practitioner visibility of the patient and their home set-up, increased knowledge about the functioning of the client, and mitigated difficulties patients had using the online platform due to age or health status. Participants identified that when OT practitioners are unable to do hands-on interventions with clients, they must have the ability to effectively coach their clients and client champions through intervention sessions. Hall et al. (2021) revealed that physical therapists who used telehealth during the COVID-19 pandemic felt that the success of the session was heavily impacted by the therapist's ability to implement a coaching model to empower caregivers. These findings support the idea that therapists rely on coaching and communication skills to overcome the barriers of the virtual environment. Previous researchers found that OT practitioners who implemented occupation-based coaching into their telehealth practices built more self-efficacy within clients and their caregivers (Little et al., 2018). The success of OT services delivered via telehealth therefore can rely heavily on the skill of the practitioner to act as a coach, resulting in the ability of the client and client champion to take increased ownership of their own progress.

OT practitioners have the capacity to adjust their communication and physical demonstration approach to match the needs of the client through a virtual platform. Flexibility of the OT practitioner is important to overcome challenges from logistical circumstances, like technical difficulties. Being flexible and client-centered helps to build the rapport between therapist and client creating a strong foundation for successful use of telehealth (Hines et al, 2019). Participants in a study conducted by Hoel et al. (2021) indicated that transitioning to telehealth services required increased flexibility initially, but after the initial adjustment period, found high levels of satisfaction with telehealth-delivered services. This supports our findings that effective communication skills and flexibility are important contributions to client safety and success of the session.

Practitioners found success with the delivery of OT interventions via telehealth with the utilization of effective coaching to guide clients. Areas of successful telehealth implementation that practitioners noted were ADL in pediatric populations, home-based ADL, education across all populations, as well as therapeutic exercise and therapeutic activity across all populations. Some OT practitioners have found increased success in areas of ADL and home-based occupations due to the session taking place in the client's natural environment. Modifications or grading of activities needed for a successful and safe completion of an ADL session in the home are more easily implemented when the OT practitioner can see the home environment. A study by Renda and Lape (2018) supports that home modification interventions delivered via telehealth can increase safety, functional performance, and how clients perceive their own performance of desired activities. Intervention styles and approaches implemented by the OT using telehealth are more successful when client champions are engaged, effective coaching of the client and client champions is implemented, and logistics allow continuous access to services.

The purpose of this study was to investigate OT practitioners, who were novice telehealth users during the COVID-19 pandemic, and their perceptions of telehealth as a delivery method for OT services. The main findings identified four themes including logistics of telehealth practice, role of client champions, capacity of the OT practitioner, and styles and approaches. Practitioners' perceptions indicated that telehealth as a delivery method for OT services increased access to populations and was most successful when client champions were engaged, practitioners were flexible and able to grade interventions based on virtual circumstances, and effective coaching and communication was used. As the use of telehealth in the field of OT continues to expand, it is important to continue research and spread knowledge related to effective service delivery in this format.

## Limitations and Implications for Practice

There is potential for implicit negative biases from participants as the practitioners surveyed in this study experienced the implementation of OT practice via telehealth in the context of high stress due to the global COVID-19 pandemic. Additionally, due to the nature of the online survey format, qualitative responses tended to be more categorical in comparison to phenomenological discussion that is typical of data collected via focus groups or interview-based research. Of note, this study intentionally focused on novice telehealth users' perspectives, however, future research into OT practitioner perspectives may benefit from the inclusion of participants with a wider variety of experience using telehealth.

OT practitioners shared important perceptions of their experiences as novel telehealth users. Overall, the findings of this study reveal that telehealth as a delivery method for OT services is supported by intentional preparation by the practitioner, flexibility, effective client coaching skills, and education. Intentional preparation by the OT practitioner before sessions can support effectiveness of services delivered and minimize logistical challenges that may arise. Aside from preparation, flexibility is also vital to overcoming the limitations of the virtual environment. OT practitioners' ability to act as a coach to their clients and client champions supports engagement, empowerment, and effective delivery of services. Implementation of education for OT practitioners regarding effective coaching methods would support practitioners' successful use of telehealth. OT education and continuing education may better prepare practitioners to deliver services via telehealth by fostering coaching skills to empower clients and client champions.
